# Lipid feature and late onset ≥80 years of ischemic stroke: a retrospective study in lipid-lowering therapy-naive adults

**DOI:** 10.3389/fneur.2026.1786974

**Published:** 2026-05-15

**Authors:** Shusheng Jiao, Yiyang Li, Xiaohong Yuan, Xiaofang Cheng

**Affiliations:** 1 Department of Neurology, Bethune International Peace Hospital, Shijiazhuang, Hebei, China; 2 Department of Geriatrics, Jiangbin Hospital of Guangxi Zhuang Autonomous Region, Nanning, Guangxi Zhuang Autonomous Region, China; 3 Department of Neurology, Dazu District Hospital of Traditional Chinese Medicine, Chongqing, China

**Keywords:** acute ischemic stroke, late onset, lipid profile, non-high-density lipoprotein cholesterol, oldest-old

## Abstract

**Background:**

The population of patients with first-onset ischemic stroke at or above the age of 80 is steadily growing. However, the distinct lipid profile characteristics of this oldest-old cohort remain systematically undefined. This study aimed to delineate these lipid patterns and elucidate their association with the late onset of acute ischemic stroke in advanced age.

**Methods:**

We conducted a retrospective cohort analysis of patients with first-ever acute ischemic stroke consecutively enrolled between March 2022 and June 2025. To mitigate confounding by lipid-lowering therapies, a critical modifier of both lipid metabolism and cerebrovascular risk, we implemented strict exclusion criteria for any prior exposure to cholesterol-modulating agents. Participants were stratified into three age groups: youngest-old (60–69 years), middle-old (70–79 years), and oldest-old (≥80 years). Demographic characteristics and lipid parameters were systematically analyzed across groups, with correlation and multivariate regression analyses performed to identify lipid-related predictors of late-onset ischemic stroke.

**Results:**

A total of 565 patients were included in the final analysis based on our inclusion and exclusion criteria (239 youngest-old; 200 middle-old; 126 oldest-old). The oldest-old group exhibited significantly lower levels of total cholesterol, triglycerides, apolipoprotein B, low-density lipoprotein cholesterol (LDL-C), very low-density lipoprotein cholesterol (VLDL-C), non-high-density lipoprotein cholesterol (non-HDL-C), and reduced LDL-C/HDL-C and VLDL-C/HDL-C ratios compared to younger cohorts (all *p* < 0.05). Additionally, this group demonstrated decreased prevalence rates of dyslipidemia subtypes, particularly for total cholesterol, triglycerides, LDL-C, and VLDL-C abnormalities. Notably, 11 distinct patterns of combined dyslipidemia showed diminished frequency in the oldest-old. Multivariate analysis identified non-HDL-C as the strongest lipid predictor of late-onset ischemic stroke in the overall population, with gender-specific variations: LDL-C emerged as the primary contributor in females, whereas triglycerides showed predominant association in males.

**Conclusion:**

The findings suggest a distinct lipid profile in older adults with late-onset ischemic stroke. A key implication for management is that non-HDL-C should be elevated to a co-primary target alongside LDL-C in public health strategies for lipid control.

## 1 Introduction

The global population aged ≥80 years is growing rapidly, projected to triple from 143 million in 2019 to 426 million by 2050 (1). This group carries a disproportionate stroke burden: ~30% of incident strokes, one-third of acute care use, and nearly 60% of 30-day stroke mortality (2). Ischemic stroke accounts for 88.27% of cases, with an annual incidence >17.23 per 1,000 person-years in this population (3), making primary prevention a cornerstone of healthy aging (4).

Aging disrupts lipid homeostasis, increasing vulnerability to cerebrovascular disease (5). Lipid markers including LDL-C, non-HDL-C, lipoprotein(a), and ApoE are established predictors of ischemic stroke (6–9). However, landmark cohort studies [Framingham (10), REGARDS (11), and China-PAR (12)] have critical limitations: most enroll predominantly younger or middle-aged adults, underrepresent nonagenarians/centenarians, and rarely exclude participants taking statins or other lipid-lowering drugs. As a result, the age-specific lipid — stroke relationship in the treatment-naive oldest-old remains poorly defined, limiting tailored prevention strategies.

To address this gap, we performed a hospital-based retrospective cohort study of first-ever acute ischemic stroke patients aged ≥60 years (2022–2024), excluding those with prior lipid-lowering therapy to avoid confounding effects on both physiological lipid alterations and the decline in cerebrovascular risk in aging. Patients were stratified into 60–69, 70–79, and ≥80 years groups. We compared baseline lipid profiles across strata and used multivariable regression to estimate age-specific associations between dyslipidemia and late-onset ischemic stroke, with validation via sensitivity analyses. This study provides unique evidence on lipid predictors in the extreme-aged population, filling key knowledge gaps for geriatric stroke prevention.

## 2 Materials and methods

### 2.1 Study population and study design

A hospital-based retrospective cohort study was conducted at our Department of Neurology, involving consecutive admissions of patients with first-ever acute ischemic stroke between March 2022 and June 2025. The cohort, which was derived from a previous study (13) with entirely different aims was stratified into three age-defined groups according to the index stroke onset age: oldest-old (≥80 years), middle-old (70–79 years), and youngest-old (60–69 years). The informed consent was waived because of the retrospective nature of the study, and the ethical approval was obtained from Bethune International Peace Hospital (2023-KY-121). All methods and procedures were carried out in accordance with relevant guidelines and regulations.

Eligibility required fulfillment of the following criteria: (1) age ≥ 60 years at stroke onset; (2) first clinical presentation of acute ischemic cerebrovascular event; (3) time-to-admission window ≤7 days from symptom onset; (4) acute cerebral infarction verified by diffusion-weighted imaging (DWI) on brain magnetic resonance imaging (MRI) performed within 24 h of admission; and (5) capacity to undergo standardized neurovascular evaluations, such as brain MRI/MRA (magnetic resonance angiography) or CTA (computed tomography angiography), cervical Doppler ultrasonography, and comprehensive laboratory profiling.

Participants were excluded for any of the following: (1) prior lipid-modifying therapy, including documented use of statins, PCSK9 inhibitors, fibrates, or other hypolipidemic agents; (2) cerebrovascular history including previous stroke (ischemic/hemorrhagic), transient ischemic attack (TIA), or neurovascular interventions; and (3) comorbid intracranial abnormalities including traumatic brain injury sequelae, neoplastic lesions or arteriovenous malformations, cerebral venous sinus thrombosis, hydrocephalus, demyelinating disorders (e.g., multiple sclerosis), or congenital malformations.

### 2.2 Collection of basic data

Comprehensive demographic and clinical parameters were systematically documented for all participants, encompassing baseline characteristics including chronological age, biological sex, current smoking status, and major comorbid conditions (atrial fibrillation, diabetes mellitus, hypertension, dyslipidemia, coronary artery disease, and hyperhomocysteinemia), along with detailed pre-admission pharmacotherapy records of antiplatelet agents, oral anticoagulants, statins, antihypertensive regimens, and glucose-lowering medications. Stroke-specific metrics were rigorously captured, comprising admission National Institutes of Health Stroke Scale (NIHSS) scores quantifying neurological deficits, precise symptom onset-to-hospitalization intervals, and etiological subclassification according to the Trial of ORG 10172 in Acute Stroke Treatment (TOAST) criteria, ensuring multidimensional characterization of acute ischemic stroke profiles across the cohort.

### 2.3 Collection of lipid data

Fasting blood samples were obtained within 24 h after emergency department visit or neurology admission. Fasting serum lipid profiles were quantitatively analyzed using standardized enzymatic assays on a Roche Cobas c702 automated biochemical analyzer, with measurements encompassing total cholesterol (TC), triglycerides (TG), low-density lipoprotein cholesterol (LDL-C), high-density lipoprotein cholesterol (HDL-C), very-low-density lipoprotein cholesterol (VLDL-C), apolipoproteins A (ApoA), B (ApoB), E (ApoE), and lipoprotein(a) [Lp(a)] within 48 h post-admission. All assays adhered to nationally validated reference intervals established for Chinese adult populations: TC < 5.18 mmol/L, TG < 1.70 mmol/L, HDL-C 1.16—1.42 mmol/L, LDL-C < 3.37 mmol/L, VLDL-C < 0.77 mmol/L, ApoA 1.20—1.60 g/L, ApoB 0.60—1.40 g/L, ApoE 2.7—4.9 mg/dL, and Lp(a) < 300 mg/L. The concentration of non-HDL-C was calculated by subtracting HDL-C from TC.

### 2.4 Statistical methods

All analyses were conducted using SPSS 18.0 (IBM Corp.) and Microsoft Office Excel 2007, with statistical significance defined as two-tailed *p* < 0.05. Continuous parameters underwent normality assessment via the Kolmogorov–Smirnov test, with subsequent characterization as mean ± standard deviation (SD) for Gaussian distributions or median with interquartile range (IQR) for nonparametric data. Categorical variables were expressed as absolute frequencies and percentages. Intergroup comparisons employed ANOVA with Tukey post-hoc correction for normally distributed continuous variables or Kruskal-Wallis tests for non-normal distributions, while categorical analyses utilized χ^2^ tests or Fisher’s exact tests as appropriate. Ordinal trends across age strata were evaluated using the Cochran-Armitage trend test.

To address potential confounding from biological sex, conventional cerebrovascular risk factors (hypertension, diabetes, etc.), and pre-stroke medications, partial correlation analyses with covariance adjustment were performed to isolate lipid-first-onset age relationships. Rank-order associations between lipid parameters and age cohorts were further quantified through Kendall’s tau-b concordance analysis. Linear regression modeling progressed through hierarchical stages: univariable analysis of lipid-age relationships preceded multivariable adjustments - Model 1 (sex + risk factors) and Model 2 (Model 1 + pre-stroke medications). A stepwise selection algorithm (entry *p* < 0.05, retention *p* < 0.10) subsequently identified dominant lipid predictors from the full parameter set. Sex-stratified analyses were conducted to account for established sexual dimorphism in both lipid metabolism and stroke pathophysiology.

## 3 Results

### 3.1 The selection of the study population

Between March 2022 and June 2025, a consecutive cohort of 2,557 patients presenting with acute ischemic stroke was initially enrolled at the Stroke Center of our Hospital. Following rigorous screening, 367 individuals were primarily excluded due to first-onset age <60 years, with an additional 918 excluded for documented stroke history (transient ischemic attack, ischemic/hemorrhagic stroke) or uncertain cerebrovascular history. Further exclusions comprised: 223 cases non-compliant with MRI-DWI protocols; 16 with motion-degraded neuroimaging; 12 lacking complete imaging data; 312 without fasting lipid profiles; 112 receiving lipid-modifying therapy; and 35 harboring structural/comorbid neurological pathologies (3 chronic subdural hematomas, 7 cavernous hemangiomas, 4 arteriovenous malformations, 1 venous sinus thrombosis, 4 hydrocephalus, 9 Alzheimer’s dementia, 1 mitochondrial encephalopathy, 1 suspected substance-related encephalopathy, 2 cerebral autosomal dominant arteriopathy suspects). The final analytical cohort comprised 565 lipid-naïve, first-ever ischemic stroke patients aged ≥60 years, stratified by index event age into three gerontological subgroups: youngest-old (60–69 years, *n* = 239), middle-old (70–79 years, *n* = 200), and oldest-old (≥80 years, *n* = 126).

### 3.2 Characteristics of basic data

Table 1 delineates the demographic and clinical characteristics of the entire cohort and stratified age groups. Consistent with epidemiological patterns, males predominated in overall first-onset ischemic stroke incidence (60.5% in total population). This gender disparity exhibited a gradual attenuation with advancing age at first onset, though male predominance persisted even in the oldest-old stratum (*p* for trend = 0.078). The oldest-old cohort demonstrated clinically significant elevations in cardiovascular comorbidities, manifesting higher prevalence of atrial fibrillation (*p* < 0.001) and coronary heart disease (*p* = 0.002). Pharmacological management patterns revealed age-dependent escalation of antiplatelet (*p* value for trend<0.001) and anticoagulant therapies (*p* value for trend = 0.007). Stroke severity quantified by NIHSS scores showed progressive augmentation across age strata (*p* value for trend<0.001). Notably, smoking prevalence exhibited an inverse relationship with advancing age (current smokers: *p* = 0.023, *p* value for trend = 0.007). TOAST subtype distribution demonstrated significant intergroup heterogeneity (*p* < 0.001) without discernible linear age-related progression (*p* value for trend = 0.417).

**Table 1:** Basic data of participants.

Variables	Youngest-old (*n* = 239)	Middle-old (*n* = 200)	Oldest-old (*n* = 126)	*p* value	*p* for trend
Demographic factors					
Age, year, median, (IQR)	65.0 (62.0–67.0)	74.0 (71.0–76.0)	84.0 (81.75–88.0)	-	-
Male sex, *n* (%)	158 (66.1)	120 (60.0)	64 (50.8)	0.017	0.078
Vascular risk factors					
Atrial fibrillation, *n* (%)	10 (4.2)	9 (4.5)	26 (20.6)	<0.001	<0.001
Diabetes mellitus, *n* (%)	87 (36.4)	71 (35.5)	41 (32.5)	0.760	0.652
Arterial hypertension, *n* (%)	154 (64.4)	126 (63.0)	85 (67.5)	0.713	0.652
Coronary heart disease, *n* (%)	33 (13.8)	34 (17.0)	41 (32.5)	<0.001	<0.001
Hyperhomocysteinemia, *n* (%)	42 (17.6)	37 (18.5)	21 (16.7)	0.913	0.887
Current smoking, *n* (%)	33 (13.8)	16 (8.0)	7 (5.6)	0.023	0.007
Previous medication, *n* (%)					
Antiplatelets	43 (18.0)	34 (17.0)	42 (33.3)	0.001	0.003
Oral anticoagulants	5 (2.1)	5 (2.5)	10 (7.9)	0.010	0.009
Antihypertensives	135 (56.5)	104 (52.0)	76 (60.3)	0.333	0.664
Antidiabetics	74 (31.0)	59 (29.5)	35 (27.8)	0.815	0.524
Clinical parameters, median, (IQR)					
NIHSS at admission	2.0 (1.0–3.0)	2.0 (1.0–4.0)	3 (1.0–5.0)	<0.001	0.009
Time onset to admission, hour	24 (12.0–48.0)	24.0 (12.0–48.0)	24.0 (12.0–48.0)	0.622	0.094
TOAST classification, *n* (%)				<0.001	0.417
Large-artery atherosclerosis	109 (45.6)	90 (45)	43 (34.1)		
Cardioembolism	5 (2.1)	3 (1.5)	21 (16.7)		
Small-artery occlusion	104 (43.5)	83 (41.5)	51 (40.5)		
Other determined etiology	8 (3.3)	6 (3.0)	2 (1.6)		
Undetermined etiology	13 (5.4)	18 (9.0)	9 (7.1)		

IQR, interquartile range; NIHSS, National Institutes of Health Stroke Scale; TOAST, Trial of ORG 10172 in Acute Stroke Treatment.

### 3.3 Age-stratified lipid profile dynamics and dyslipidemia pattern evolution

**Figure 1:**
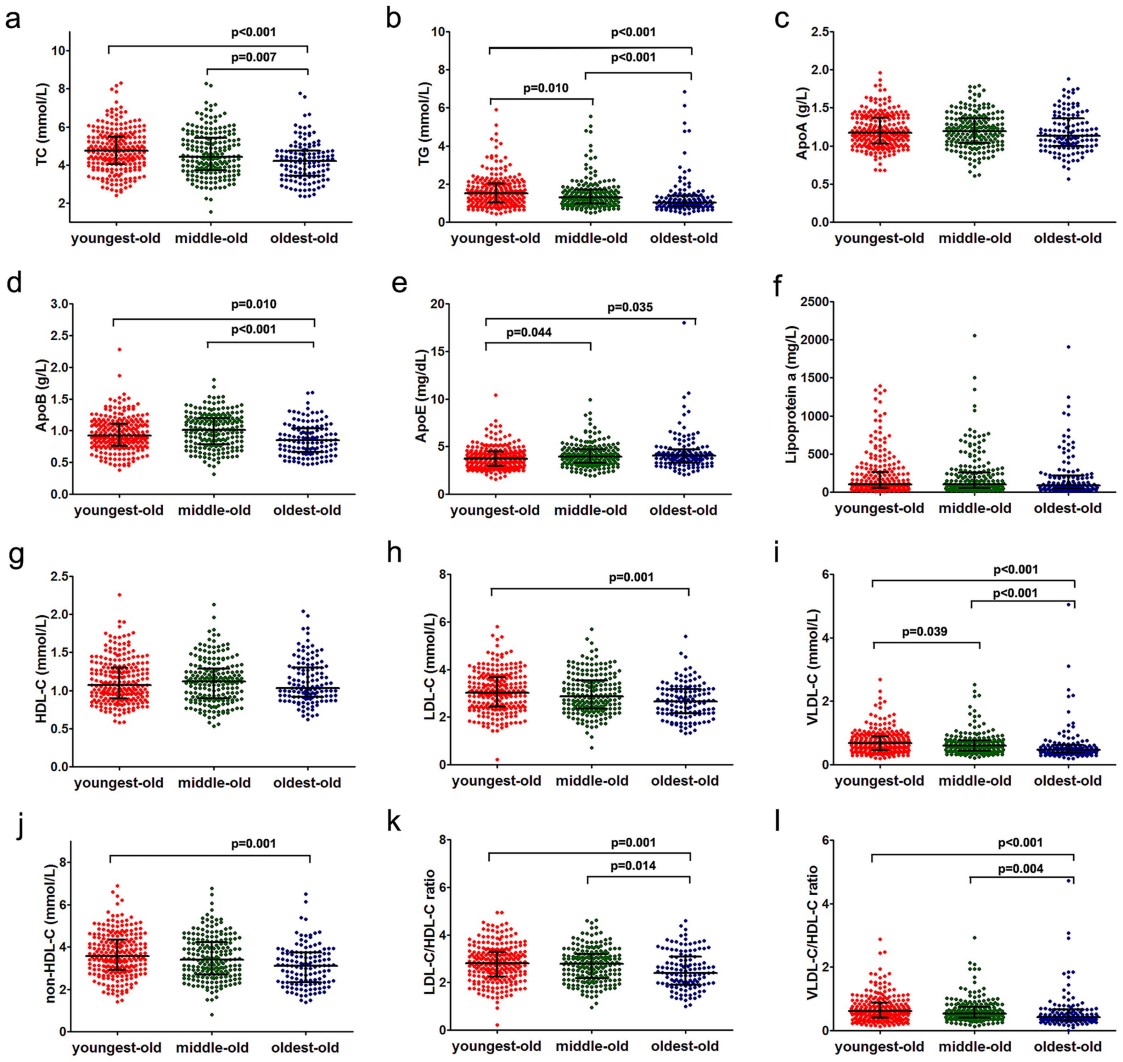
Age-stratified variations in lipid fractions across youngest-old, middle-old, and oldest-old cohorts. Panels **(a)–(l)** respectively show the distribution, mean values, standard deviations, and intergroup statistical comparisons (via ANOVA) of the following parameters among the three patient groups: TC, TG, ApoA, ApoB, ApoE, lipoprotein(a), HDL-C, LDL-C, VLDL-C, non-HDL-C, LDL-C/HDL-C ratio, and VLDL-C/HDL-C ratio. Non-significant intergroup comparisons (*p* ≥ 0.05) were not annotated in the graphical representations.

The analytical framework categorized lipid parameters into primary lipid fractions (TC, TG, ApoA, ApoB, ApoE, lipoprotein(a), HDL-C, LDL-C, VLDL-C) and calculated ratios (non-HDL-C, LDL-C/HDL-C, VLDL-C/HDL-C, ApoB/ApoA). Median concentrations (or values) with interquartile ranges (IQR) for fasting lipids are detailed in Table 2. Among primary variables, age-stratified comparisons revealed significant intergroup differences in TC, TG, ApoB, ApoE, LDL-C, and VLDL-C (all *p* < 0.05). The oldest-old cohort demonstrated progressive reductions in TC, TG, LDL-C, and VLDL-C, alongside elevated ApoE levels, relative to younger groups (Figure 1). For derived indices, non-HDL-C, LDL-C/HDL-C, and VLDL-C/HDL-C exhibited inverse associations with age, while ApoB/ApoA showed no significant trend (Figure 1).

**Table 2:** Measured fasting levels of 9 lipid species and 4 derived ratios.

Variables, median, (IQR)	Youngest-old (*n* = 239)	Middle-old (*n* = 200)	Oldest-old (*n* = 126)	*p* value
TC (mmol/L)	4.74 (4.04–5.49)	4.43 (3.72–5.42)	4.22 (3.43–4.75)	0.001
TG (mmol/L)	1.52 (1.03–2.02)	1.29 (0.96–1.70)	1.03 (0.83–1.38)	<0.001
ApoA (g/L)	1.17 (1.03–1.37)	1.20 (1.04–1.36)	1.13 (1.00–1.36)	0.377
ApoB (g/L)	0.92 (0.76–1.11)	1.01 (0.78–1.20)	0.85 (0.67–1.04)	<0.001
ApoE (mg/dL)	3.74 (2.96–4.50)	3.96 (3.31–4.68)	4.04 (3.31–4.71)	0.012
Lipoprotein a (mg/L)	100.30 (55.93–265.95)	100.60 (53.52–259.48)	91.60 (49.85–213.4)	0.712
HDL-C (mmol/L)	1.07 (0.90–1.30)	1.12 (0.90–1.29)	1.03 (0.92–1.30)	0.062
LDL-C (mmol/L)	3.02 (2.43–3.67)	2.88 (2.36–3.54)	2.66 (2.15–3.18)	0.001
VLDL-C (mmol/L)	0.68 (0.46–0.89)	0.60 (0.44–0.77)	0.47 (0.38–0.63)	<0.001
Non-HDL-C (mmol/L)	3.58 (2.92–4.35)	3.39 (2.69–4.23)	3.12 (2.33–3.74)	0.001
LDL-C/HDL-C ratio	2.80 (2.25–3.29)	2.28 (2.18–3.20)	2.40 (1.90–3.08)	0.001
VLDL-C/HDL-C ratio	0.61 (0.41–0.87)	0.54 (0.40–0.74)	0.43 (0.31–0.67)	<0.001
ApoB/ApoA ratio	0.78 (0.64–0.95)	0.84 (0.68–1.02)	0.75 (0.56–0.93)	0.003

IQR, interquartile range; TC, total cholesterol; TG, triglycerides; ApoA, apolipoprotein A; ApoB, apolipoprotein B; ApoE, apolipoprotein E; HDL, high-density lipoprotein cholesterol; LDL-C, low-density lipoprotein cholesterol; VLDL, very low-density lipoprotein cholesterol.

**Figure 2:**
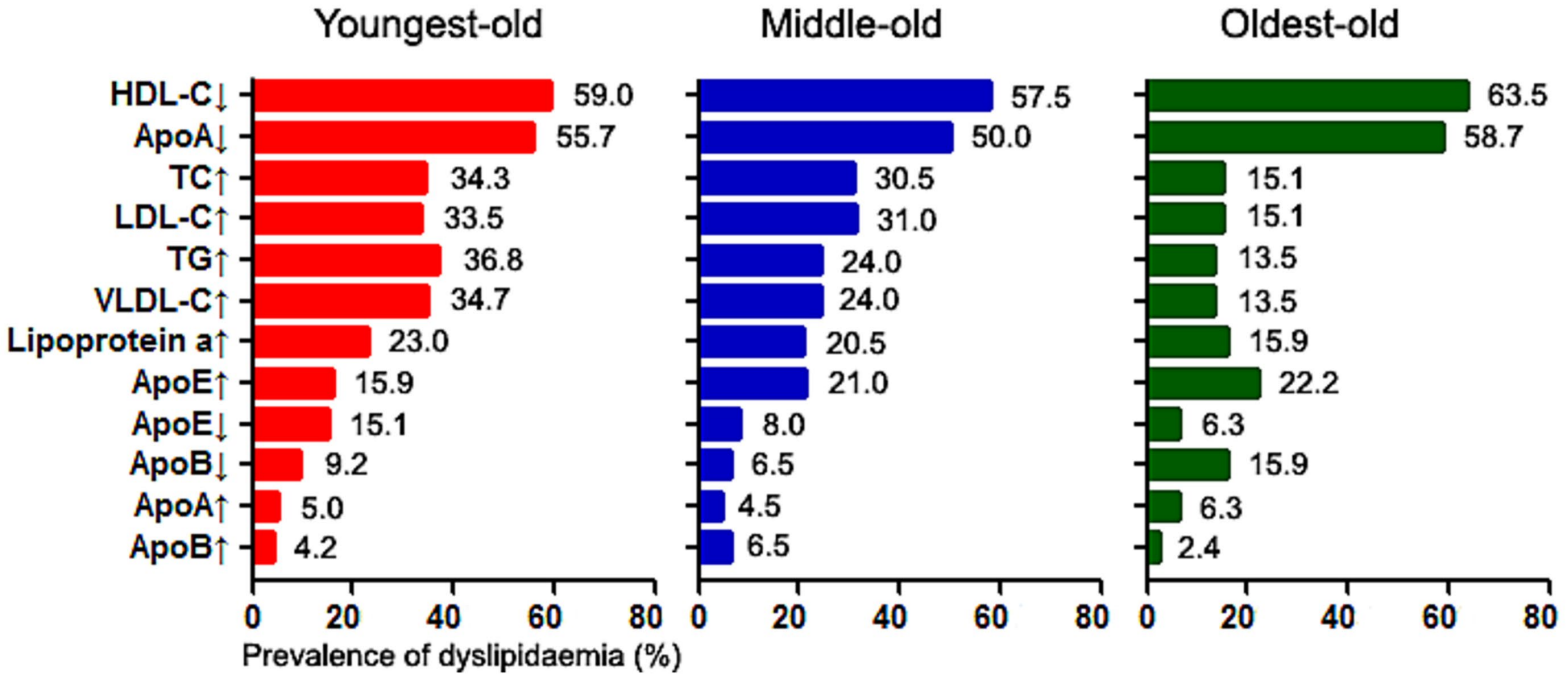
Age-dependent reconfiguration of isolated dyslipidemia subtypes: prevalence ranking shifts in single-parameter lipid abnormalities with advancing stroke onset age.

Dyslipidemia prevalence and subtypes are summarized in Table 3 and Supplementary Table 1. In the total cohort, single-parameter abnormalities predominated as follows: low HDL-C (59.5%), hypo-ApoA (54.3%), hyper-TC (28.7%), hyper-LDL-C (28.5%), hyper-TG (27.1%), elevated VLDL-C (26.2%), hyper-lipoprotein(a) (20.5%), hyper-ApoE (19.1%), hypo-ApoE (10.6%), hypo-ApoB (9.7%), hyper-ApoA (5.1%), and hyper-ApoB (4.6%). Age-dependent shifts in abnormality rankings were observed (Figure 2), with hyper-TC, hyper-LDL-C, hyper-TG, and hyper-VLDL-C demonstrating positive age associations (all *p* value <0.05, all *p* value for trend <0.01), whereas hypo-ApoE prevalence declined in the oldest-old (*p* value for trend = 0.01).

**Table 3:** Age-stratified prevalence of single-parameter dyslipidemia.

Variables	Youngest-old (*n* = 239)	Middle-old (*n* = 200)	Oldest-old (*n* = 126)	*p* value	*p* for trend
HDL-C↓, *n* (%)	141 (59.0)	115 (57.5)	80 (63.5)	0.552	0.495
ApoA↓, *n* (%)	133 (55.7)	100 (50.0)	74 (58.7)	0.264	0.795
TC↑, *n* (%)	82 (34.3)	61 (30.5)	19 (15.1)	<0.001	<0.001
LDL-C↑, *n* (%)	80 (33.5)	62 (31.0)	19 (15.1)	0.001	0.001
TG↑, *n* (%)	88 (36.8)	48 (24.0)	17 (13.5)	<0.001	<0.001
VLDL-C↑, *n* (%)	83 (34.7)	48 (24.0)	17 (13.5)	<0.001	<0.001
Lipoprotein a↑, *n* (%)	55 (23.0)	41 (20.5)	20 (15.9)	0.276	0.110
ApoE↑, *n* (%)	38 (15.9)	42 (21.0)	28 (22.2)	0.241	0.111
ApoE↓, *n* (%)	36 (15.1)	16 (8.0)	8 (6.3)	0.012	0.005
ApoB↓, *n* (%)	22 (9.2)	13 (6.5)	20 (15.9)	0.020	0.122
ApoA↑, *n* (%)	12 (5.0)	9 (4.5)	8 (6.3)	0.758	0.660
ApoB↑, *n* (%)	10 (4.2)	13 (6.5)	3 (2.4)	0.207	0.643

IQR, interquartile range; TC, total cholesterol; TG, triglycerides; ApoA, apolipoprotein A; ApoB, apolipoprotein B; ApoE, apolipoprotein E; HDL, high-density lipoprotein cholesterol; LDL-C, low-density lipoprotein cholesterol; VLDL, very low-density lipoprotein cholesterol.

**Figure 3:**
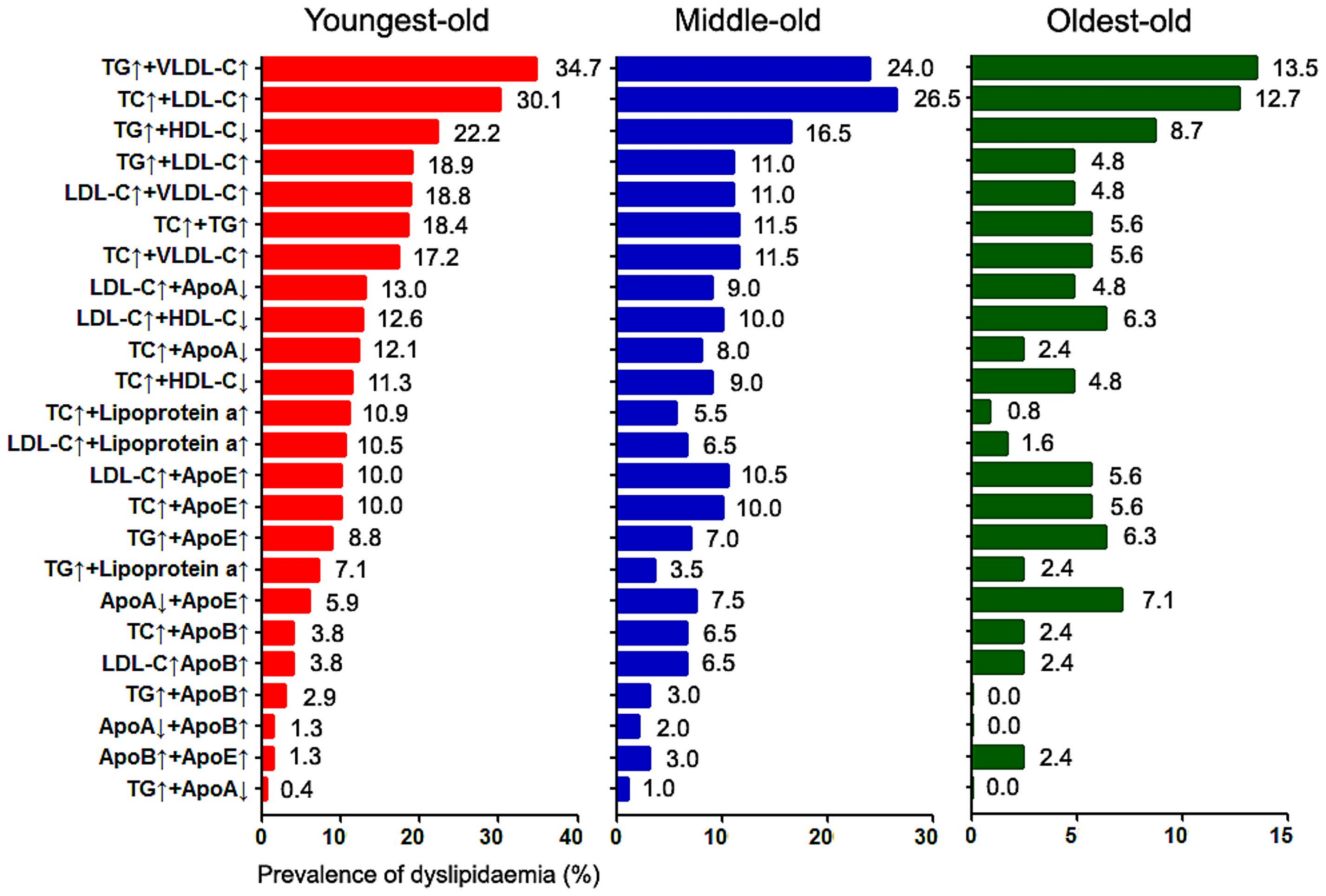
Age-dependent reconfiguration of multifactorial dyslipidemia: prevalence ranking shifts in two-parameter lipid abnormalities with advancing stroke onset age.

For multi-parameter dyslipidemia (≥2 variables), the five most prevalent combinations in the overall cohort were: TG + VLDL-C (26.2%), TC + LDL-C (25.0%), TG + HDL-C (17.2%), TC + TG (13.1%), and TG + LDL-C (12.9%), with age-related ranking variations (Figure 3). Eleven distinct dual-parameter subtypes showed significant frequency differences across age strata (Supplementary Table 1), all exhibiting reduced prevalence in the oldest-old group (range: *p* = 0.038 to *p* < 0.001). Among mixed dyslipidemias (≥3 variables), the four most frequent types displayed stable intergroup rankings but demonstrated declining rates of TC + TG + LDL-C and TC + TG + lipoprotein(a) with advancing age.

### 3.4 Correlation of lipid parameters with advancing age strata

Kendall’s tau-b analysis identified eight lipid parameters demonstrating significant associations with advancing age strata (Table 4). Inverse correlations were observed for TC, TG, LDL-C, VLDL-C, non-HDL-C, LDL-C/HDL-C ratio and VLDL-C/HDL-C ratio, while ApoE exhibited a positive correlation. Notably, partial correlation analysis adjusting for covariates including sex, conventional cerebrovascular risk factors, and pre-existing medication use revealed an additional inverse relationship between ApoB levels and first-onset age. This expanded the spectrum of age-associated lipid parameters to nine clinically relevant biomarkers.

**Table 4:** Correlation of lipid parameters with age group and first-onset age.

Variables	Age groups (Kendall test)*		First-onset age (partial correlation test)^#^	
	Correlation coefficient	*p* value	Correlation coefficient	*p* value
TC	−0.141	<0.001	−0.207	<0.001
TG	−0.187	<0.001	−0.160	<0.001
ApoA	−0.028	0.388	−0.079	0.064
ApoB	−0.056	0.088	−0.103	0.015
ApoE	0.094	0.004	0.114	0.007
Lipoprotein a	−0.026	0.433	0.008	0.857
HDL-C	−0.011	0.744	−0.041	0.330
LDL-C	−0.124	<0.001	−0.186	<0.001
VLDL-C	−0.178	<0.001	−0.127	0.003
Non-HDL-C	−0.154	<0.001	−0.219	<0.001
LDL-C/HDL-C ratio	−0.111	0.001	−0.141	0.001
VLDL-C/HDL-C ratio	−0.155	<0.001	−0.100	0.018
ApoB/ApoA ratio	−0.017	0.611	−0.039	0.358

IQR, interquartile range; TC, total cholesterol; TG, triglycerides; ApoA, apolipoprotein A; ApoB, apolipoprotein B; ApoE, apolipoprotein E; HDL, high-density lipoprotein cholesterol; LDL-C, low-density lipoprotein cholesterol; VLDL, very low-density lipoprotein cholesterol. *The Kendall correlation analysis was performed as the three age groups followed the characteristics of rank variable. ^#^The partial correlation test was performed with adjustments for sex, vascular risk factors and previous medications in order to eliminate the influence of other possible factors on this relationship.

### 3.5 Lipid determinants of late-onset stroke in the oldest-old

To further elucidate the predictive capacity of lipid profiles on late-onset ischemic stroke in extreme age, we conducted hierarchical linear regression analyses using age at first stroke onset as a continuous variable to quantify late-onset predisposition. The analytical framework comprised three sequential models: unadjusted univariate associations; adjusted for sex and conventional stroke risk factors (model 1); and further adjusted for pre-stroke medication exposure (model 3). The results were shown in Table 5. Univariate analysis identified TC, TG, LDL-C, non-HDL-C, and LDL-C/HDL-C ratio as principal lipid determinants of late onset (all *p* < 0.001). These associations persisted through multivariable adjustments, maintaining consistent effect directions and magnitudes in models 1–2. Secondary predictors including ApoB, ApoE, VLDL-C, and VLDL-C/HDL-C ratio demonstrated significant contributions across all models (*p* < 0.05). Stepwise multivariate regression ultimately retained non-HDL-C as the predominant lipid predictor in the total cohort, with sex-specific stratification revealing LDL-C dominance in females versus TG predominance in males.

**Table 5:** The effect of lipid profile on first-onset age (linear regression analysis).

Variables	Univariable analysis		Multivariable analysis (model 1)*		Multivariable analysis (model 2)^#^	
	OR vales	*p* value	OR (95% CI)	*p* value	OR (95% CI)	*p* value
TC	−1.568 (−2.182–-0.955)	<0.001	−1.642 (−2.248–-1.036)	<0.001	−1.519 (−2.117–-0.920)	<0.001
TG	−1.650 (−2.463–-0.836)	<0.001	−1.650 (−2.451–-0.850)	<0.001	−1.553 (−2.356–-0.751)	<0.001
ApoA	−2.638 (−5.699–0.423)	0.091	−3.782 (−6.918–-0.646)	0.018	−2.836 (−5.839–0.168)	0.064
ApoB	−3.652 (−6.274–-1.031)	0.006	−3.297 (−5.859–-0.735)	0.012	−3.181 (−5.745–-0.617)	0.015
ApoE	0.716 (0.213–1.220)	0.005	0.641 (0.012–0.143)	0.012	0.667 (0.180–1.154)	0.007
Lipoprotein a	0.000 (−0.002–0.003)	0.946	0.000 (0.753–-0.002)	0.753	0.000 (−0.002–0.003)	0.857
HDL-C	−0.869 (−3.376–1.638)	0.496	−1.778 (−4.333–0.777)	0.172	−1.218 (−3.672–1.237)	0.330
LDL-C	−1.986 (−2.822–-1.149)	<0.001	−2.006 (−2.831–-1.181)	<0.001	−1.860 (−2.679–-1.040)	<0.001
VLDL-C	−2.627 (−4.288–-0.966)	0.002	−2.720 (−4.353–-1.087)	0.001	−2.513 (−4.148–-0.878)	0.003
Non-HDL-C	−1.873 (−2.553–-1.193)	<0.001	−1.867 (−2.533–-1.200)	<0.001	−1.788 (−2.452–-1.124)	<0.001
LDL-C/HDL-C ratio	−1.785 (−2.706–-0.864)	<0.001	−1.532 (−2.435–-0.629)	0.001	−1.546 (−2.453–-0.639)	0.001
VLDL-C/HDL-C ratio	−2.103 (−3.681–-0.525)	0.009	−1.953 (−3.491–-0.415)	0.013	−1.872 (−3.422–-0.322)	0.018
ApoB/ApoA ratio	−1.595 (−4.162–-0.972)	0.223	−0.958 (0.452–-3.460)	0.452	−1.173 (−3.678–1.331)	0.358

IQR, interquartile range; TC, total cholesterol; TG, triglycerides; ApoA, apolipoprotein A; ApoB, apolipoprotein B; ApoE, apolipoprotein E; HDL, high-density lipoprotein cholesterol; LDL-C, low-density lipoprotein cholesterol; VLDL, very low-density lipoprotein cholesterol. *Model 1was adjusted for sex, risk factors; ^#^Model 2 was adjusted for sex, risk factors and previous medications.

## 4 Discussion

The exponential growth of the geriatric population necessitates heightened focus on cerebrovascular disorders in advanced age, particularly given the distinct pathophysiological profile of first-ever ischemic stroke in octogenarians and beyond compared to younger elderly subgroups (14). This demographic divergence underscores the critical need for cohort-specific investigations to optimize preventive neurology strategies. Identification of late-onset stroke predictors through routine clinical biomarkers holds particular translational value, combining practical accessibility with prognostic potential. Nevertheless, research targeting the oldest-old remains constrained by either insufficient sample representation or systematic exclusion from clinical trials (15). Our study specifically addresses this gap through meticulous characterization of *de novo* stroke occurrence in extreme longevity.

Within neurovascular pathophysiology, lipids serve dual roles as structural components maintaining blood–brain barrier integrity and dynamic regulators of cerebral perfusion (16). While dyslipidemia constitutes a well-established modifiable stroke risk factor (RR 1.8–2.6), contemporary guidelines advocate expanded lipidomic profiling beyond conventional parameters for enhanced risk stratification (17). Paradoxically, despite the ubiquity of fasting lipid panels in clinical practice and their prominence in cardiocerebrovascular research (18), their predictive utility for incident stroke in advanced aging remains underexplored. This investigation systematically evaluates this relationship, incorporating critical methodological considerations frequently overlooked in prior studies. Notably, our analytical approach rigorously accounts for confounding by lipid-lowering therapies - a crucial refinement given statins’ pleiotropic neuroprotective properties extending beyond LDL-C reduction. The Northern Manhattan Study exemplifies this complexity (19), demonstrating medication status fundamentally modulates LDL-C’s stroke risk association. By restricting analysis to statin-naïve individuals, we mitigate therapeutic confounding while clarifying baseline lipid-stroke relationships in untreated elderly populations.

This investigation provides a multidimensional characterization of dyslipidemia patterns in statin-naïve octogenarians and beyond presenting with incident ischemic stroke. First, we delineated age-dependent trajectories of 9 core lipid fractions (TC, TG, ApoB, LDL-C, VLDL-C, etc.) and 4 composite indices under medication-free conditions - a critical perspective scarcely addressed in prior geriatric lipidomics research. Second, our dual-axis analysis integrating quantitative levels and qualitative abnormality frequencies revealed distinct geroprotective lipid signatures: the extreme-age cohort exhibited attenuated atherogenic profiles (hypo-TC, TG, ApoB, LDL-C, VLDL-C, non-HDL-C, LDL-C/HDL-C and VLDL-C/HDL-C, relative to younger groups) with concomitant hypo-prevalence of dyslipidemia subtypes. These findings collectively suggest that early-onset stroke in lipidotherapy-free elderly reflects multifactorial dysregulation, necessitating precision prevention frameworks combining comprehensive lipid panel monitoring with individualized threshold optimization. Third, through integrative trend, correlation, and hierarchical regression analyses, we established non-HDL-C as the predominant lipid determinant of stroke chronobiology in extreme aging. Non-HDL-C’s predictive superiority stems from its atherogenic lipoprotein spectrum inclusion - beyond LDL-C, it quantifies triglyceride-rich lipoproteins (TRL), remnant particles, and lipoprotein(a), all implicated in statin-resistant residual stroke risk (20, 21). Our findings corroborate emerging consensus positioning non-HDL-C as an independent cerebrovascular risk biomarker (22), particularly advantageous in elderly populations due to: fasting state independence enhancing clinical utility; superior predictive validity in metabolic syndrome phenotypes prevalent among elderly populations (obesity, diabetes, metabolic disorders, etc); stronger mortality correlation in high-risk demographics (23). These evidence-based advantages advocate for dual targeting of non-HDL-C and LDL-C in geriatric lipid guidelines, potentially revolutionizing primary stroke prevention strategies through enhanced residual risk capture.

Our findings suggest that maintaining suboptimal non-HDL-C levels may confer cerebroprotective benefits by delaying ischemic stroke onset in geriatric populations. Effective management of atherogenic lipid burden requires multimodal intervention combining lifestyle modifications (dietary optimization, structured exercise regimens) with pharmacotherapy. Current evidence confirms that while statins - the cornerstone of lipid-lowering therapy - significantly reduce LDL-C concentrations, their impact on non-HDL-C remains moderate, highlighting persistent residual cardiovascular risk (24). Emerging clinical trial data present promising alternatives: PCSK9 inhibitors demonstrate enhanced efficacy in non-HDL-C reduction through dual LDL receptor upregulation and VLDL particle clearance; similarly, high-dose omega-3 formulations and next-generation fibrates show synergistic effects, achieving superior non-HDL-C reductions (28–34%) compared to conventional therapies. Nevertheless, critical knowledge gaps persist regarding: optimal non-HDL-C thresholds for extreme-age cohorts; long-term safety profiles of combination therapies; pleiotropic effects beyond lipid modulation. These unresolved questions underscore the imperative for targeted therapeutic development, particularly novel pharmacologic agents addressing non-HDL-C components like remnant cholesterol and lipoprotein(a) - key drivers of residual stroke risk in statin-treated elderly patients.

Our sex-stratified analysis revealed a distinct pathophysiological dichotomy in lipid-stroke associations. Male geriatric patients demonstrated higher predisposition to atherogenic dyslipidemia characterized by hypertriglyceridemia, whereas female counterparts exhibited greater susceptibility to isolated LDL-C dysregulation. These findings may be related to estrogen-mediated LDL receptor regulation and sex differences in adipose tissue lipolysis, respectively. Nevertheless, these results require further confirmation through large-scale, multicenter randomized controlled trials.

Unlike these earlier investigations that primarily focused on younger older adults (aged 65–75 years), our study provides novel data specifically for the oldest-old (≥80 years) who were lipid-lowering therapy-naive, thereby eliminating confounding by chronic medication use. Notably, direct comparisons between our study and previous cohorts are limited by differences in study design (retrospective vs. prospective), age distribution, and, most importantly, the exclusion of lipid-lowering therapy users in our study—a feature that distinguishes our work but also limits direct generalizability. Future prospective, multicenter studies with standardized lipid measurement protocols are needed to validate our findings across diverse ethnic and geographic populations.

## 5 Limitations

Several methodological constraints warrant consideration. First, the single-center design inherently limits generalizability, necessitating external validation across diverse ethnic and geographic populations to confirm our observed lipid-stroke chronobiology associations. Second, exclusion of silent cerebrovascular events (SCIs) introduces temporal bias, as subclinical neuropathology preceding clinical stroke onset may alter true lipid-risk temporal dynamics. This omission potentially distorts estimations of atherogenic lipid burden’s temporal effects. Most critically, the application of static lipid thresholds across aging strata contravenes established geriatric lipid metabolism trajectories. Current diagnostic frameworks lack age-adjusted cut-offs validated for octogenarian and beyond populations, particularly regarding triglyceride-rich lipoprotein thresholds. This standardization gap, acknowledged in recent ESC/EAS (European Society of Cardiology/European Atherosclerosis Society) guidelines, may confound interpretation of dyslipidemia-stroke relationships in extreme longevity cohorts. We note that in the majority of included patients, fasting blood samples were obtained within 12 h after the first dose of statin therapy was administered. Although it is generally accepted that a reduction in LDL-C becomes detectable only after 3–5 days of treatment, the potential influence of short-term statin exposure on baseline lipid measurements cannot be completely ruled out. Given that lipid levels are dynamic and influenced by multiple factors, future studies should ideally analyze lipid profiles derived from the three most recent health examination reports prior to stroke onset to minimize bias and enhance the validity of the findings. Furthermore, conditions that substantially influence lipid metabolism — such as hepatic and renal dysfunction, thyroid disorders, malignancies, and chronic wasting diseases — were not explicitly excluded from our study. This may have introduced residual confounding, and future studies should incorporate systematic screening or statistical adjustment for these factors to validate our findings.

## 6 Conclusion

While dyslipidemia is an established cerebrovascular risk factor, its temporal relationship with late-onset ischemic stroke in geriatric populations (≥80 years) remains poorly characterized. This study provides the first comprehensive characterization of age-specific lipid abnormalities and quantifies their chronobiological contributions to stroke manifestation in extreme longevity. Our findings advance understanding of geriatric stroke pathophysiology and inform targeted lipid management strategies for this rapidly expanding demographic.

## Data Availability

The datasets presented in this study can be found in online repositories. The names of the repository/repositories and accession number(s) can be found in the article/Supplementary material.
