# Supplementary material for: Lipid feature and late onset ≥80 years of ischemic stroke: a retrospective study in lipid-lowering therapy-naive adults
Source: Front Neurol. 2026 May 15;17:1786974. doi: 10.3389/fneur.2026.1786974 (PMC13235004; doi:10.3389/fneur.2026.1786974)
Supplement: Supplementary file 1 [file Table_1.docx]

**Supplementary Information**

**Supplementary table 1. Frequencies of dyslipidaemia involving at least two or three values in three age groups**

| **Variables** | **Youngest-old (n =239)** | **Middle-old (n =200)** | **Oldest-old (n =126)** | ***P* value** |
| --- | --- | --- | --- | --- |
| Two values combined |  |  |  |  |
| TC↑ + TG↑, n(%) | 44(18.4) | 23(11.5) | 7(5.6) | 0.002 |
| TC↑+HDL-C↓, n(%) | 27(11.3) | 18(9.0) | 6(4.8) | 0.117 |
| TC↑+LDL-C↑, n(%) | 72(30.1) | 53(26.5) | 16(12.7) | 0.001 |
| TC↑+VLDL-C↑, n(%) | 41(17.2) | 23(11.5) | 7(5.6) | 0.005 |
| TC↑+Lipoprotein a↑, n(%) | 26(10.9) | 11(5.5) | 1(0.8) | 0.001 |
| TC↑+ApoA ↓, n(%) | 29(12.1) | 16(8.0) | 3(2.4) | 0.006 |
| TC↑+ApoB↑, n(%) | 9(3.8) | 13(6.5) | 3(2.4) | 0.171 |
| TC↑+ApoE↑, n(%) | 24(10.0) | 20(10.0) | 7(5.6) | 0.304 |
| TG↑+HDL-C↓, n(%) | 53(22.2) | 33(16.5) | 11(8.7) | 0.005 |
| TG↑+LDL-C↑, n(%) | 45(18.9) | 22(11.0) | 6(4.8) | 0.000 |
| TG↑+VLDL-C↑, n(%) | 83(34.7) | 48(24.0) | 17(13.5) | 0.000 |
| TG↑+Lipoprotein a↑, n(%) | 17(7.1) | 7(3.5) | 3(2.4) | 0.075 |
| TG↑+ApoA ↓, n(%) | 1(0.4) | 2(1.0) | 0(0.0) | 0.458 |
| TG↑+ApoB↑, n(%) | 7(2.9) | 6(3.0) | 1(0.8) | 0.386 |
| TG↑+ApoE↑, n(%) | 21(8.8) | 14(7.0) | 8(6.3) | 0.650 |
| LDL-C↑+HDL-C↓, n(%) | 30(12.6) | 20(10.0) | 8(6.3) | 0.176 |
| LDL-C↑+VLDL-C↑, n(%) | 45(18.8) | 22(11.0) | 6(4.8) | 0.000 |
| LDL-C↑+Lipoprotein a↑, n(%) | 25(10.5) | 13(6.5) | 2(1.6) | 0.007 |
| LDL-C↑+ApoA↓, n(%) | 31(13.0) | 18(9.0) | 6(4.8) | 0.038 |
| LDL-C↑+ApoB↑, n(%) | 9(3.8) | 13(6.5) | 3(2.4) | 0.171 |
| LDL-C↑+ApoE↑, n(%) | 24(10.0) | 21(10.5) | 7(5.6) | 0.271 |
| ApoA↓+ApoB↑, n(%) | 3(1.3) | 4(2.0) | 0(0.0) | 0.283 |
| ApoA↓+ApoE↑, n(%) | 14(5.9) | 15(7.5) | 9(7.1) | 0.774 |
| ApoB↑+ApoE↑, n(%) | 3(1.3) | 6(3.0) | 3(2.4) | 0.439 |
| Three values combined, n(%) |  |  |  |  |
| TC↑+TG↑+LDL-C↑, n(%) | 41(17.2) | 19(9.5) | 5(4.0) | 0.000 |
| TC↑+TG↑+HDL-C↓, n(%) | 17(7.1) | 12(6.0) | 3(2.4) | 0.172 |
| TC↑+TG↑+Lipoprotein a↑, n(%) | 12(5.0) | 4(2.0) | 0(0.0) | 0.015 |
| TC↑+LDL-C↑+HDL-C↓, n(%) | 22(9.2) | 15(7.5) | 5(4.0) | 0.193 |

**Abbreviations**: IQR, interquartile range; TC, total cholesterol; TG, triglycerides; ApoA, apolipoprotein A; ApoB, apolipoprotein B; ApoE, apolipoprotein E; HDL, high-density lipoprotein cholesterol; LDL-C, low-density lipoprotein cholesterol; VLDL, very low-density lipoprotein cholesterol.
